# Prospective Study of Antibiotic Prophylaxis for Prostate Biopsy Involving >1100 Men

**DOI:** 10.1100/2012/650858

**Published:** 2012-05-02

**Authors:** Rustom P. Manecksha, Gregory J. Nason, Ivor M. Cullen, Jérôme P. Fennell, Elizabeth McEvoy, Ted McDermott, Robert J. Flynn, Ronald Grainger, John A. Thornhill

**Affiliations:** ^1^Department of Urology, Adelaide and Meath Hospital, Tallaght, Dublin 24, Ireland; ^2^Department of Microbiology, Adelaide and Meath Hospital, Tallaght, Dublin 24, Ireland

## Abstract

We aimed to compare infection rates for two 3-day antibiotic prophylaxis regimens for transrectal ultrasound-guided prostate biopsy (TRUSgbp) and demonstrate local microbiological trends. In 2008, 558 men and, in 2009, 625 men had TRUSgpb. Regimen 1 (2008) comprised 400 mg Ofloxacin immediately before biopsy and 200 mg 12-hourly for 3 days. Regimen 2 (2009) comprised Ofloxacin 200 mg 12-hourly for 3 days commencing 24 hours before biopsy. 20/558 (3.6%) men had febrile episodes with regimen 1 and 10/625 (1.6%) men with regimen 2 (*P* = 0.03). *E. coli* was the most frequently isolated organism. Overall, 7/13 (54%) of positive urine cultures were quinolone resistant and (5/13) 40% were multidrug resistant. Overall, 5/9 (56%) patients with septicaemia were quinolone resistant. All patients were sensitive to Meropenem. There was 1 (0.2%) death with regimen 1. Commencing Ofloxacin 24 hours before TRUSgpb reduced the incidence of febrile episodes significantly. We observed the emergence of quinolone and multidrug-resistant *E. coli*. Meropenem should be considered for unresolving sepsis.

## 1. Introduction

Prostate cancer remains a leading cause of cancer mortality in many Western countries [1, 2]. In the Irish context, commencement of the National Cancer Control Programme for prostate cancer will undoubtedly result in an increase in men having transrectal ultrasound-guided prostate biopsy (TRUSgpb) and being diagnosed with prostate cancer. The diagnosis of prostate cancer is mainly made on TRUSgpb, which is commonly performed and safe but is associated with a small risk of infective complications including symptomatic bacteriuria, bacteraemia, and potentially life-threatening sepsis [3]. Antibiotic prophylaxis is the accepted best practice for patients undergoing TRUSgpb but there is a lack of consensus regarding the optimum regimen [4–6]. Bacteriuria after TRUSgpb is decreased with antibiotic prophylaxis compared with placebo [7]. Burden et al. highlight the lack of standardized antibiotic guidelines and the general lack of evidence-based practice for TRUSgpb [8].

Although many antimicrobial agents are used in practice, quinolones remain the most widely used antibiotics due to their broad spectrum activity, ease of oral administration, good penetration of prostate tissue, and long-lasting urinary bactericidal activity. The infection rate with antibiotic prophylaxis is estimated at 5% and rate of septicaemia about 0.5–1% [9, 10]. Infective complications are similar when comparing 1-day versus 3-day ciprofloxacin [11, 12] but guidelines acknowledge that the choice of regimens remains debatable [6].

We compared the rate of infective complications of our standard antibiotic prophylaxis regimen with that of a modified regimen. We also report on the current microbiological flora and antibiotic resistance in our region.

## 2. Methods

From January 2008 to December 2009, we prospectively recruited 1183 consecutive men who had TRUSgpb indicated by an elevated serum prostate-specific antigen level and/or a prostatic abnormality on digital rectal examination. All patients gave consent for inclusion. Our standard antibiotic prophylaxis protocol comprised three days of oral Ofloxacin 200 mg 12-hourly, starting with a loading dose of 400 mg oral Ofloxacin administered immediately before TRUSgpb (regimen 1). In 2008, 558 consecutive men had TRUSgpb with regimen 1. In 2009, 625 consecutive men had TRUSgpb with a modified regimen, comprising three days of 200 mg Ofloxacin 12-hourly commencing 24 hours before TRUSgpb (regimen 2). All patients had 8–12 core biopsies using 18G needles after injecting 5–10 ml of 1% lignocaine local anaesthetic. Following TRUSgpb, patients were given verbal and written instructions to attend the nearest medical facility if they experienced fevers, rigors, or symptoms suggestive of a urinary infection. Every patient returned to our department for biopsy results two weeks after TRUSgpb, and every patient was asked if they had presented to another medical facility with infection, ensuring even patients who presented to other medical facilities were included. Urine and blood culture results and antibiotic sensitivities were obtained from the respective laboratories. Urine samples were cultured on CLED agar and CHROMagar according to accredited protocols. Organisms were identified using either CHROMagar colony colour or VITEK-2 (bioMérieux, France). Blood cultures were incubated using the BacT Alert (bioMérieux, France) system. Any significant cultures were identified using API (bioMérieux, France) or VITEK-2 biochemical testing methods. Susceptibility testing was done according to Clinical Laboratory Standards Institute criteria for all significant isolates. A febrile episode was defined as an illness accompanied by temperature >37.5°C. Multidrug resistance was defined as resistance to three or more antibiotic classes [13]. The *t*-test was used for statistical analysis. The criterion for statistical significance was a *P* < 0.05.

## 3. Results

1183 men had TRUSgpb, 558 men with regimen 1 (2008) and 625 men with regimen 2 (2009). Patient demographics were homogeneous for both groups. Mean age of patients was comparable between the two groups, 60 years for regimen 1 and 64 years for regimen 2 (*P* = 0.3). Mean number of biopsy cores was also comparable for both regimens, 9 for regimen 1 and 10 for regimen 2 (*P* = 0.99). All patients were Caucasian. The rate of repeat biopsies was comparable between the two regimens with 84 repeat biopsies (15%) for regimen 1 and 98 repeat biopsies (16%) for regimen 2 (*P* = 0.8). 20/558 (3.6%) men had febrile episodes with regimen 1 compared with 10/625 (1.6%) with regimen 2 (*P* = 0.03), a relative risk reduction of 55% with regimen 2 (odds ratio = 0.44). With regimen 1, of the 20 febrile episodes, 8 (40%) men had positive urine cultures and 6 (30%) men had positive blood cultures. 4/8 (50%) men with positive urine cultures also had positive blood cultures. With regimen 2, of the 10 febrile episodes, 6 (60%) men had positive urine cultures and 4 (40%) men had positive blood cultures. 3/6 (50%) men with positive urine cultures also had positive blood cultures. With regimen 1, *Escherichia coli* (*E. coli*) was isolated in 100% of men with positive urine cultures, 5/8 (63%) of which were quinolone-resistant. 2/8 (25%) *E. coli* isolates were multidrug-resistant. Antibiotic resistance for *E. coli* is shown in Tables 1 and 2. In 2009, *E. coli* were isolated in 5/6 (83%) men and *Enterococcus* was isolated in 1/6 men with positive urine cultures. Multidrug-resistant *E. coli* strains including one extended spectrum beta-lactamase (ESBL) producer were isolated in 2/5 (40%) men. The one ESBL-producing *E. coli* strain was multi-drug resistant and sensitive only to amikacin and Meropenem. 6/558 (1.1%) men had septicaemia with regimen 1, compared with 4/625 (0.64%) men with regimen 2 but this was not statistically significant (*P* = 0.4). *E. coli* was isolated in blood culture in 5/6 (83%) men with septicaemia with regimen 1, of which 4/5 (80%) were quinolone-resistant; *Enterococcus* was isolated from the sixth man. *E. coli* was isolated in 100% of men with septicaemia with regimen 2, of which 1/4 (25%) was quinolone resistant. All were sensitive to Meropenem. There was one multidrug-resistant *E. coli* sepsis-related mortality with regimen 1 (0.2%) and none with regimen 2.

## 4. Discussion

Although the rates of febrile episodes and bacteraemia in both our groups of men are similar to other series, rates for regimen 2 compared more favourably [3, 10, 14, 15]. Of all patients with febrile episodes, 30–60% of our patients had positive urine cultures or blood cultures, again, similar to other reports [3, 16].

Although those men with febrile episodes may have presented to other medical facilities, all patients returned to our department for their biopsy results two weeks after biopsy. At that consultation, patients were asked specifically about any visits or admissions to other medical facilities and data was then obtained from those facilities. Therefore, we are confident that our data accurately reflects the incidence of infection in our department.

There is no doubt that antibiotic prophylaxis has significantly decreased the infectious complications associated with TRUSgpb. Nonetheless, infective complications still occur, albeit rarely, and can be potentially life threatening, as reflected by the death in our department in 2008.

While many urologists use quinolones for antibiotic prophylaxis prior to TRUSgpb, there is a lack of consensus as to the choice of antibiotic, dose, and duration of prophylaxis. Suggested regimens include single-dose fluoroquinolone to a 3-day course starting either immediately before biopsy or a day before biopsy [6, 8, 17, 18]. Multidrug-resistant and quinolone-resistant *E. coli* are increasingly prevalent in various countries with up to 20–30% resistance reported in England, Spain, the USA, and Taiwan [15, 19–21]. Because of geographical variation in microbiological flora, local bacterial prevalence and resistance profiles are necessary to facilitate specific tailoring of antibiotic prophylaxis regimens. The need to individualize regimens to the microbiological patterns of each region, perhaps, contributes to the lack of consensus of the subject.

The differing infection rates between our two regimens are probably explained by the pharmacokinetics and bioavailability of oral Ofloxacin, which reaches peak plasma concentration 1.5–2 hours after administration [22]. If bacterial seeding occurs during or immediately after biopsy, then peak plasma concentration of antibiotic is necessary at the time of biopsy. With regimen 1, where the first dose was immediately before biopsy, peak plasma concentration would be subtherapeutic at the time of biopsy. With regimen 2, commencing Ofloxacin 24 hours prior to biopsy, peak plasma concentration would have been achieved at the time of biopsy, explaining the reduction in the rate of infection and septicaemia. Nonetheless, 3/4 patients with septicaemia in regimen 2 were quinolone sensitive (Table 2), which on retrospective questioning was established to be due to lack of compliance. Peak plasma concentration for Ofloxacin is reached within two hours; therefore, commencing prophylaxis two hours before biopsy would have been ideal. However, we anticipated problems with compliance with instructions to commence antibiotic prophylaxis two hours prior to TRUSgpb; therefore, we elected to commence prophylaxis 24 hours before biopsy.

In addition to quinolone resistance, we observed resistance to aminoglycosides (Gentamicin and Amikacin, Tables 1 and 2), traditionally regarded as the antibiotic of choice for gram-negative infections. Third-generation cephalosporins showed low levels of resistance and would represent a good first-line choice. We accept that firm conclusions about changing microbiological flora cannot be drawn from this study; however, the spectrum and prevalence of antibiotic resistance is of interest and importance.

In this study, our patients were not screened for risk factors, for example, previous antibiotic exposure, previous hospital admissions or surgery, recent foreign travel, infections, in particular, urinary tract infections, obesity, diabetes mellitus, and residence in nursing homes [23]. Risk profiling may identify patients who require additional or alternative prophylaxis, and we have since commenced a further study screening for risk factors. Although, in this study, there was only one culture positive for ESBL *E. coli*, ESBL infections and multidrug resistance have been recognized as a growing worldwide problem in the community and in hospitals. The high frequency of multidrug resistance among ESBL-producing strains greatly limits the possibilities of administering an adequate prophylactic regimen to these patients [24]. As prevalence increases, it may be necessary to introduce additional risk reduction measures, for example, rectal swabs [25] to screen for pathogens including ESBL prior to TRUSgpb to allow better antibiotic selection for these patients. This prospective study was not randomised and nonblinded, which the authors accept as a limitation. However, the authors feel the prospective consecutive nature of recruitment meant there was no selection bias. The biopsy technique, number of cores, and patient population were comparable for both groups eliminating further confounders. A further limitation is the small number of febrile adverse events. However, the total number of patients who had TRUSgpb is large and the authors feel that this data provides useful insight into the microbiological profile of our region and adds to the existing data on emerging global trends. The authors accept that data now exists showing a single preoperative dose of fluoroquinolone to be comparable to 3-day regimens [12]; however, many units still employ a short course for prophylaxis and there is a lack of overall consensus on the most appropriate regimen.

The commonest bacteria isolated in our region were *E. coli*. Commencing Ofloxacin prophylaxis 24 hours prior to TRUS biopsy was associated with fewer febrile and septicaemic episodes, although the latter was not statistically significant. Quinolone and multidrug-resistant *E. coli* are emerging among our patients. The overall rate of infection and septicaemia is low and TRUSgpb remains a safe procedure. However, the microbiological trends are striking and important. Given the overall low incidence of febrile episodes and septicaemia with Ofloxacin prophylaxis, quinolones remain a good choice of antibiotic for prophylaxis; however, clinicians should consider possible resistance in febrile patients following TRUSgpb.

## 5. Conclusion

It is important for centres to be aware of local microbiological trends and antibiotic resistance. When treating a septic patient following-TRUSgpb, we recommend intravenous third-generation cephalosporin, for example, ceftriaxone or cefotaxime as first-line therapy (Table 2). Consideration should be given to using Meropenem for sepsis unresponsive to first-line intravenous antibiotics.

## Figures and Tables

**Table 1:** Antibiotic resistance for *E. coli* isolated in urine cultures.

Antibiotic resistance (%)	Regimen 1 (*n* = 8)	Regime 2 (*n* = 5)	Total (*n* = 13)
Amoxicillin	5 (63%)	2 (40%)	7 (54%)
Coamoxiclav	3 (38%)	1 (20%)	5 (38%)
Ofloxacin	5 (63%)	2 (40%)	7 (54%)
Gentamicin	2 (25%)	2 (40%)	4 (31%)
Amikacin	2 (25%)	1 (20%)	3 (23%)
3rd-generation cephalosporin^§^	0 (0%)	1 (20%)	1 (8%)
Meropenem	0 (0%)	0 (0%)	0 (0%)

^§^3rd-generation cephalosporin either ceftriaxone or cefotaxime.

**Table 2:** Antibiotic resistance for *E. coli* isolated in blood cultures.

Antibiotic resistance (%)	Regimen 1 (*n* = 5)	Regime 2 (*n* = 4)	Total (*n* = 9)
Amoxicillin	3 (60%)	3 (75%)	6 (67%)
Coamoxiclav	1 (20%)	2 (50%)	3 (33%)
Ofloxacin	4 (80%)	1 (25%)	5 (56%)
Gentamicin	2 (40%)	1 (25%)	3 (33%)
Amikacin	2 (40%)	0 (0%)	2 (22%)
3rd-generation cephalosporin^§^	0 (0%)	0 (0%)	0 (0%)
Piperacillin/Tazobactam	0 (0%)	0 (0%)	0 (0%)
Meropenem	0 (0%)	0 (0%)	0 (0%)

^§^3rd-generation cephalosporin either ceftriaxone or cefotaxime.
